# Solid-Pseudopapillary Tumor of the Pancreas: A Single Center Experience

**DOI:** 10.1155/2016/4289736

**Published:** 2016-12-29

**Authors:** Valentina Beltrame, Gioia Pozza, Enrico Dalla Bona, Alberto Fantin, Michele Valmasoni, Cosimo Sperti

**Affiliations:** ^1^Department of Surgery, Oncology and Gastroenterology, 3rd Surgical Clinic, University of Padua, Via Giustiniani 2, 35128 Padua, Italy; ^2^Gastroenterology Unit, University of Padua, Via Giustiniani 2, 35128 Padua, Italy

## Abstract

Aim of this study was to review the institutional experience of solid-pseudopapillary tumors of the pancreas with particular attention to the problems of preoperative diagnosis and treatment. From 1997 to 2013, SPT was diagnosed in 18 patients among 451 pancreatic cystic neoplasms (3.7%). All patients underwent preoperative abdominal ultrasound, computed assisted tomography, and tumor markers (CEA and CA 19-9) determinations. In some instances, magnetic resonance, positron emission tomography, and endoscopic ultrasound with aspiration cytology were performed. There were two males and 16 females. Serum CA 19-9 was slightly elevated in one case. Preoperative diagnosis was neuroendocrine tumor (*n* = 2), mucinous tumor (*n* = 2), and SPT (*n* = 14). Two patients underwent previous operation before referral to our department: one explorative laparotomy and one enucleation of SPT resulting in surgical margins involvement. All patients underwent pancreatic resection associated with portal vein resection (*n* = 1) or liver metastases (*n* = 1). One patient died of metastatic disease, 77 months after operation, and 17 are alive and free with a median survival time of 81.5 months (range 36–228 months). Most of SPT can be diagnosed by CT or MRI, and the role of other diagnostic tools is very limited. We lack sufficient information regarding clinicopathologic features predicting prognosis. Caution is needed when performing limited resection, and long and careful follow-up is required for all patients after surgery.

## 1. Introduction

Solid-pseudopapillary tumor (SPT) is a distinct variety among cystic neoplasms of the pancreas. Although rare, this type of tumor is increasingly seen in clinical practice because of widespread availability of imaging modalities and better awareness of the disease. In a review of English literature from 1933 to 2003, a total of 718 SPTs were collected, including pediatric cases [1]; in a recent review of Law et al. in 2014 [2], a total of 2744 patients with SPT were identified, of whom 2410 were observed from 2000 to 2012. Solid-pseudopapillary tumor is generally considered an indolent lesion with low malignant potential; it occurs most frequently in young women. Favorable prognosis after surgical resection has been invariably reported. However, some cases of locally infiltrating or metastatic variety, or recurrences after surgery, have been described in a significant percentage of 10–15% of patients [3]. Long-term survival is reported even for metastatic disease, but some patients will eventually die for disease's progression, suggesting a widely variable and not clearly elucidated biology of the tumor.

We report our experience of SPTs with particular attention to identify the problems in differential diagnosis, the clinicopathological features predicting behaviour, and the treatment and outcome after tumor's resection.

## 2. Patients and Methods

We retrospectively reviewed the patients who underwent surgical resection of SPT of the pancreas from the prospectively recorded database, between January 1997 and December 2013. We evaluated patients' demographic features, clinical presentation, imaging findings, surgical procedures, pathologic aspects, perioperative outcome, follow-up, and survival. All patients preoperatively underwent serum carcinoembryonic antigen CEA and CA 19-9 examination, abdominal ultrasonography (US), and computed assisted tomography (CT). In some instances, magnetic resonance imaging (MRI), 18-FDG positron emission tomography (PET/CT), and endoscopic ultrasound (EUS) with fine-needle aspiration (FNA) were also performed. Pathologically, SPTs were classified as malignant if it showed extrapancreatic invasion, perineural or vascular invasion, pancreatic parenchyma invasion [4], or distant metastases. Follow-up included physical examination, serum tumor markers, and US and/or CT or MRI every 6 months for the first 5 years and then every year. PET/CT was performed when clinically suggested. The median follow-up period was 84 months (range 36–228 months).

## 3. Results

**Figure 1:**
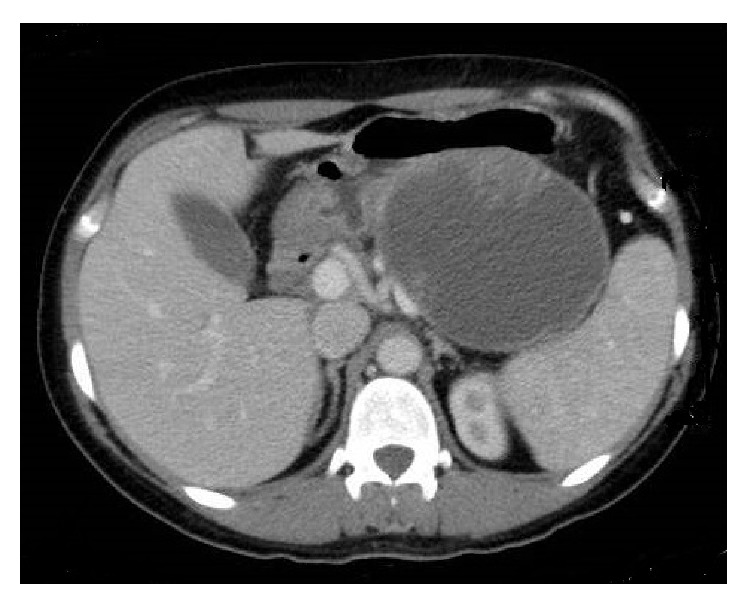
Computed tomography of the abdomen showing a large cystic mass with solid components in the body-tail of the pancreas (case number 13).

**Figure 2:**
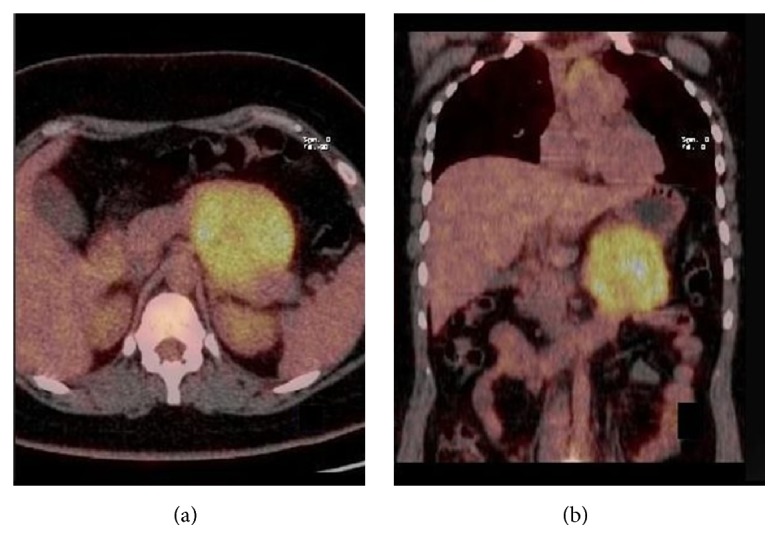
Positron emission tomography with CT acquisition (PET/CT) of the abdomen: axial (a) and coronal image (b) showing a pathologic uptake of FDG in a well-circumscribed, round mass in the tail of the pancreas (case number 15).

In the study period, 451 patients with cystic tumors of the pancreas were observed; among these, 18 (3.7%) were histologically proven to have SPT of the pancreas (Table 1). There were 16 females and 2 males, with a mean age of 34.2 years (range 13–75): both male patients were older compared to females. Patients presented abdominal pain or discomfort (*n* = 9) and palpable mass (*n* = 6); three patients were asymptomatic. The tumor averaged 7.0 cm in diameter (range 2–14 cm) and was located in the body and/or tail of the pancreas in 13 patients, in the head in 3 patients, and in the neck in two patients. Three patients had surgery in other hospitals before referral to our department: one patient had cystogastrostomy for an incorrect diagnosis of pancreatic pseudocyst; one had exploratory laparotomy and biopsy for a locally advanced pancreatic mass involving portal-mesenteric vein; and one young patient had enucleation of 3 cm pancreatic head mass that showed surgical margins involvement at pathologic examination. Only one patient showed high serum CA 19-9 levels (92 U/mL; normal value < 37 U/mL). Preoperative radiological investigation included abdominal US and CT in all patients (Figure 1), MRI in 6 patients, and EUS + fine-needle aspiration in 5. Two patients had a correct cytologic diagnosis of SPT; 2 patients had a suggested diagnosis of neuroendocrine tumor (but with radiologic findings suggestive for SPT) and in one cytology was nondiagnostic. Ten patients underwent 18-FDG positron emission tomography (PET); in 7 patients there was a pathologic uptake of the radiotracer (mean SUV 8.8, range 2.6–24.0) (Figure 2). Two patients underwent 111-In-Octreoscan without pathologic uptake of the radiotracer. So, preoperative diagnosis was neuroendocrine tumor (*n* = 2), mucinous tumor (*n* = 2), and SPT (*n* = 14). Twelve patients underwent distal pancreatectomy (4 with spleen preservation and 2 with laparoscopic approach), 3 pylorus-preserving pancreaticoduodenectomy, 2 central pancreatectomy and 1 total pancreatectomy. One patient had associated resection of involved portal vein segment with jugular vein reconstruction. The invasion of portal vein was confirmed at pathological examination but no adjuvant chemotherapy was scheduled since the patient refused any other treatment. Another patient had synchronous resection of two small hepatic metastases. The patient reoperated after enucleation of pancreatic head lesion and underwent pancreaticoduodenectomy; pathological examination showed residual SPT.

All 18 patients had R0 resection, and there were no surgical mortalities. Postoperative complications occurred in 5 patients (28%); according to the International Study Group on Pancreatic Fistula (ISGPF) [5], one patient had Grade A, and four patients had Grade B pancreatic fistula, the latter requiring drainage under radiologic guidance.

**Figure 3:**
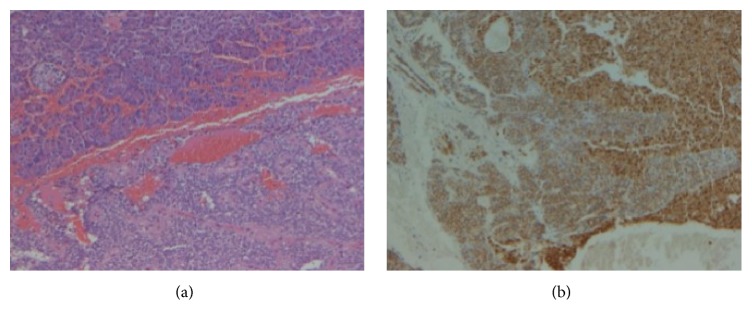
Hematoxylin and eosin stain (H&E, 100x) of SPT showing normal pancreas on the upper left side and neoplastic cells in the lower right side (a) and immunohistochemical *β*-catenin slide (100x) showing the different pattern of staining in normal pancreas (cytoplasmic) and in neoplastic pancreas (nuclear) (b).

Pathologic examination showed cellular atypia in 3 patients (numbers 5, 10, and 17), vascular invasion in 4 (numbers 5, 10, 12, and 13), perineural invasion in 4 (nr 9, 10, 16, 17), capsular invasion in 4 (numbers 3, 5, 9, and 10), and lymph node and liver metastases only in one patient (number 10). All but one patient (number 8) showed Mib 1 ≤ 1 (Table 2). B-catenin was always expressed. (Figure 3). One patient (number 10) had postoperative adjuvant therapy (gemcitabine regimen).

**Figure 4:**
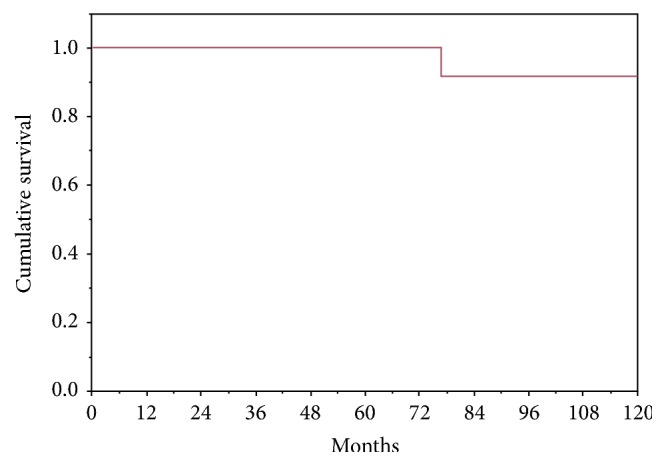
Kaplan-Meier survival curve of patients who underwent pancreatic resection of SPT.

One patient died 77 months after operation, 45 months after tumor recurrence (liver metastases); the remaining patients are alive and well, free of disease with a median survival time of 81.5 months (range 36–228 months) (Figure 4).

## 4. Discussion

Solid-pseudopapillary tumor is a very rare neoplasm of the pancreas, accounting for only 1-2% of all exocrine pancreatic tumors [3]; in our experience we observed 18 SPTs among a total of 451 (3.7%) cystic tumors of the pancreas from 1997 to 2013. In the last decade, there has been a significant increase in the number of SPTs published in the English literature [2], confirming the increasing interest toward this unique neoplasm. Most of our patients were female, at young age (mean 34.2 years, range 13–75). Only two patients were males, older than females, both are alive and free of disease for 48 and 84 months, respectively. It has been reported that male patients have distinct patterns of onset and aggressiveness compared with female patients. Machado et al. [6] observed that SPTs in male patients were more aggressive than those in female patients, and they should be managed more radically. On the contrary, Cai et al. [7]. collected 16 male patients with SPTs, and observed that males were older than female patients and had a favorable outcome after surgery, with no recurrence or death of disease in the follow-up.

Clinical presentation of SPTs is not specific. The most frequent symptoms in our series were abdominal pain or discomfort, followed by abdominal mass; 2 patients were asymptomatic. These findings are well in accordance with previous reports [7–9]. Preoperative diagnosis of SPTs is generally made by CT or MRI imaging. Typically the tumor shows a large, well circumscribed, heterogeneous mass with varying solid and cystic components, generally demarcated by a peripheral capsule and occasional calcifications [10, 11]. These findings, together with other clinical findings, such as age and sex, may be sufficient for a correct diagnosis, as in more half of our patients (Figure 1). Problems in differential diagnosis occur in the presence of small, solid lesions, or in large, unilocular cyst, or in male patients. Recently, EUS with FNA has been advocated as useful diagnostic tool also in SPTs. The diagnostic accuracy of EUS-FNA for SPT was found to be 75% in a multicentric experience of Jani et al. [12] in 2008. More recently, Law et al. [13] reported that the addition of EUS-FNA to a preoperative work-up of SPT significantly increased the diagnostic yield to 82.4%. In our experience, 5 patients underwent preoperative EUS-FNA: only 2 patients had a correct diagnosis of SPT. One patient had inconclusive results, and two had a suspected diagnosis of neuroendocrine tumor; this finding led us to perform octreotide scintigraphy and serum hormonal studies (both negative). However, CT and MR findings of these two patients were compatible with the diagnosis of SPT. In one patient EUS-FNA was complicated by mild acute pancreatitis. Recently, Virgilio et al. [14] reported a case of rupture of SPT following EUS-FNA. So the real utility of EUS in the diagnostic work-up of SPT is unclear and it appears indicated in very selected, doubtful cases.

Positron emission tomography with 18-FDG has an emerging role in the diagnosis and staging of pancreatic neoplasms, including cystic tumors [15]. The role of PET in these rare tumors is obviously not well defined. We perform preoperative PET in 10 patients: 7 showed a pathologic uptake in the tumor's area (Figure 2), while three patients did not. However, high accumulation of FDG in the tumor does not correlate with more aggressive behaviour, clinical characteristics, or histopathological features of malignancy, since all but one of these patients are alive without evidence of tumor's relapse. Dong et al. [16] studied 8 patients with SPTs who underwent preoperative PET; they found a relationship between standard uptake value (SUVmax) and histological malignancy of the tumor. However, all patients were alive without recurrence after surgery, although follow-up was very short.

Recently, Kang et al. [17] reported a large experience of 37 SPTs studied with 18-FDG PET; the pattern of FDG uptake in SPTs was not associated with histopathologic features suggestive of malignant potential. Moreover, SUVmax apparently increased according to the degree of Ki-67 expression, but without statistical significance. They concluded that the clinical usefulness of PET in SPTs needs to be further investigated. At the moment, it appears that 18-FDG PET does not have a substantial role in the diagnosis of SPTs.

In our series, only one patient presented with preoperative slight increase of serum CA 19-9, so the role of tumor markers in both the diagnosis and monitoring of the disease appears very limited.

SPT is generally considered as a tumor with low malignant potential. Although resection of the tumor provides a 5-year survival rate more than 95% [18], local recurrence or distant metastases can occur. Moreover a minority of patients show locally advanced or metastatic disease at their initial presentation. One patient presented with malignant locally advanced tumor, invading the portal vein. Total pancreatectomy with portal vein resection and reconstruction with jugular vein graft was performed, but the tumor recurred in the liver and the patient died 77 months after surgery. Locally infiltrative solid-pseudopapillary tumors of the pancreas occur infrequently; in 2008 we collected from English literature, a total of 20 patients with locally malignant SPT: 10 patients had portal vein or mesenteric vessels involvement (associated with liver metastases in two cases) and 10 had invasion of other organs (colon, spleen, kidney, adrenal gland, and omentum) [3].

A recent case series [19] of 131 consecutive resections for SPT has been published, showing only one case of metastatic disease at presentation and two cases of recurrence after resection of primary SPT. In all cases, capsular and/or pancreatic parenchyma invasion were found. No adjuvant or neoadjuvant chemotherapy has been administered. One patient eventually died because of disease progression 56 months after distal pancreatectomy. Cheng et al. [20] reported their experience of 8 patients with SPT infiltrating the portal-mesenteric vein, who underwent pancreatectomy associated with vascular resection. All but one patient were alive and free of disease after a median follow-up period of 67.5 months. One patient who underwent R1 resection died of liver recurrence 54 months after operation. So vascular invasion, although uncommon, does occur; vascular resection and reconstruction is warranted whenever possible, because long-term survival is not infrequent even in locally advanced disease.

Some reports found that a high Ki-67 mitotic index occurred more frequently in patients with malignant SPT and seems to be associated with a shorter survival [21–24]. Yang et al. [25] found that a Ki-67 ≥ 4% was significantly associated (*p* < 0.001) with recurrence. In our experience neither high Mib 1 nor Ki-67 ≥ 4% was associated with clinical or histopathological features of malignancy.

Recently, there is increasing interest in less aggressive surgical procedures for benign or border-line neoplasms of the pancreas, in order to preserve pancreatic function. So limited resection (i.e., enucleation, central pancreatectomy, spleen preservation, etc.) has been advocated also for SPTs [26]. However, one of our patients underwent pancreaticoduodenectomy for a previously enucleated SPT of the head of the pancreas that showed margin involvement at pathologic examination.

Recurrence after apparently radical resection of SPT is well reported: metastasis either at the time of presentation or, less commonly, some years after resection of the primary tumor develops in less than 15% of cases, and the liver is the most common site [18, 26].

One of our patients presented with synchronous liver metastases which were removed together with pancreaticoduodenectomy. Although there is no evidence of efficient chemotherapeutic drugs, adjuvant therapy with gemcitabine was performed, and the patient is alive, without recurrence, more than 6 years after surgery.

Resection of liver metastases is possible if the involvement of the liver is limited; survival after metastasis excision is good because of the indolent nature of the disease, and it appears that the 5-year survival is not significantly altered even in the presence of metastatic disease [18, 27]. Only occasional death from tumor, usually after many years, has been reported. Our experience confirms that, at present, there are no established clinical or histological criteria to predict the biological behaviour of SPT. While invasion of blood vessels, perineural infiltration, invasion of adjacent structures, and elevated mitotic rate are suggested to be associated with metastases and recurrence in some reports [25, 28, 29], they appear to be not related to prognosis in other experience [30], and the absence of these features does not preclude malignant behaviour [22], so long-term follow-up is warranted in all patients [31].

The utility of chemotherapy and radiotherapy for patients with SPT is substantially unknown, although there are some anecdotal reports of benefit [32–36]. Kang et al. [37] performed in vitro adenosine triphosphate based chemotherapy response assay in five resected SPT of the pancreas; cisplatin was shown to be the most effective single-chemotherapeutic agent. This finding has been reported in some previous experiences [38, 39] but not confirmed in others [40]. The limited number of reported cases explains the lack of standard treatment, and thus chemotherapeutic agents used are substantially experimental.

## 5. Conclusions

Solid-pseudopapillary tumor of the pancreas is an enigmatic tumor, mostly presenting with benign course or, less frequently, with aggressive behaviour or relapse after resection. Preoperative diagnosis is substantially made by CT or MRI in most cases. Because of its rarity, we lack sufficient information regarding clinicopathologic features predicting prognosis, and the role of radiochemotherapy in unresectable disease is clearly unknown. At moment, the biologic behaviour of SPT is unpredictable, so surgery remains the only chance of treatment even in locally or metastatic presentation. Long and careful follow-up is recommended after resection for all patients.

## Figures and Tables

**Table 1:** Clinicopathologic features of patients with pancreatic SPT.

Pts	Sex	Age	Site	Size	Treatment	Follow-up (months)
(1)	Female	54	Tail	4.0	DP	A,NED (228)
(2)	Female	13	Body	4.0	CP	A,NED (198)
(3)	Female	32	Tail	7.0	DP	A,NED (192)
(4)	Female	31	Tail	14.0	DP	A,NED (180)
(5)	Female	20	Tail	10.0	DP	A,NED (156)
(6)	Female	14	Tail	10.0	DP	A,NED (132)
(7)	Female	38	Body	3.0	DP	A,NED (96)
(8)	Male	59	Tail	11.0	DP	A,NED (94)
(9)	Female	40	Body	2.0	CP	A,NED (84)
(10)	Female	21	Head	8.0	PPPD	A,NED (79)
(11)	Female	13	Head	3.0	PPPD	A,NED (77)
(12)	Female	49	Head-body	10.0	TP + VR	DEAD (77)
(13)	Female	38	Tail	4.0	DPSP	A,NED (74)
(14)	Male	75	Tail	4.5	DP	A,NED (72)
(15)	Female	30	Tail	10.0	DP	A,NED (61)
(16)	Female	24	Body	7.0	DP	A,NED (50)
(17)	Female	14	Head	3.0	PPPD	A,NED (48)
(18)	Female	35	Tail	4.5	DP	A,NED (36)

DP = distal pancreatectomy; CP = central pancreatectomy; TP = total pancreatectomy; VR = venous resection; PPPD = pylorus-preserving pancreaticoduodenectomy; A = alive; NED = no evidence of disease; DPSP = distal pancreatectomy spleen-preserving.

**Table 2:** Clinicopathological features of Benign and Malignant SPT.

	Benign	Malignant
	(*n* = 10)	(*n* = 8)
Age		
<40	7	6
≥40	3	2
Sex		
F	8	8
M	2	0
Tumor size		
<5	6	3
≥5	4	5
Tumor localization		
Head-neck	1	3
Body-tail	9	5
R0 resection	10	8
Pancreatic parenchyma/capsular invasion	0	4
Vascular invasion	0	4
Perineural invasion	0	4
Cellular atypia	0	3
Metastases	0	1
Mib1		
<1%	9	8
≥1%	1	0
Ki-67		
<4%	10	8
≥4%	0	0
